# Ensemble Learning to Improve the Prediction of Fetal Macrosomia and Large-for-Gestational Age

**DOI:** 10.3390/jcm9020380

**Published:** 2020-01-31

**Authors:** Shangyuan Ye, Hui Zhang, Fuyan Shi, Jing Guo, Suzhen Wang, Bo Zhang

**Affiliations:** 1 Department of Population Medicine, Harvard Pilgrim Health Care Institute and Harvard Medical School, Boston, MA 02115, USA; shangyuan_ye@harvardpilgrim.org; 2 Division of Biostatistics, Department of Prevention Medicine, Northwestern University Feinberg School of Medicine, Chicago, IL 60611, USA.; hzhang@northwestern.edu; 3 School of Public Health and Management, Weifang Medical University, Weifang, Shandong 261053, China; shifuyan@wfmc.edu.cn; 4 School of Public Health, Peking University, Beijing 100191, China; jing624218@bjmu.edu.cn; 5 Department of Neurology and ICCTR Biostatistics and Research Design Center, Boston Children’s Hospital and Harvard Medical School, Boston, MA 02115, USA

**Keywords:** macrosomia, large for gestational age, machine learning, ensemble methods, prediction, sensitivity, specificity

## Abstract

Background: The objective of this study was to investigate the use of ensemble methods to improve the prediction of fetal macrosomia and large for gestational age from prenatal ultrasound imaging measurements. Methods: We evaluated and compared the prediction accuracies of nonlinear and quadratic mixed-effects models coupled with 26 different empirical formulas for estimating fetal weights in predicting large fetuses at birth. The data for the investigation were taken from the Successive Small-for-Gestational-Age-Births study. Ensemble methods, a class of machine learning techniques, were used to improve the prediction accuracies by combining the individual models and empirical formulas. Results: The prediction accuracy of individual statistical models and empirical formulas varied considerably in predicting macrosomia but varied less in predicting large for gestational age. Two ensemble methods, voting and stacking, with model selection, can combine the strengths of individual models and formulas and can improve the prediction accuracy. Conclusions: Ensemble learning can improve the prediction of fetal macrosomia and large for gestational age and have the potential to assist obstetricians in clinical decisions.

## 1. Introduction

Excessive fetal growth poses risks to maternal and infant well-being [1]. The term “macrosomia” is used to describe the condition of a fetus with a birth weight of more than 4000 g, regardless of gestational age [1,2]. Macrosomia is sometimes confused with “large for gestational age” (LGA), which describes an infant with a 90th percentile or higher birthweight for gestational age [1]. It has been shown that the infants with either macrosomia or LGA pose a large risk of perinatal morbidity and mortality to their mothers [2,3]. Therefore, accurately predicting and diagnosing these conditions has been a major goal of obstetric investigators, for purposes of conducting early intervention or targeted perinatal medical care to reduce the risks.

Measurements taken from prenatal ultrasound imaging are the primary quantitative resources for predicting birth weights and diagnosing macrosomia or LGA. Zhang et al. [4] took the empirical formula given by Hadlock et al. [5] for estimating fetal weights and implemented a joint mixed-effects model to predict macrosomia and LGA. This procedure of predicting macrosomia or LGA from prenatal ultrasound measurements was a two-step supervised learning process. In the first step, an empirical formula was chosen to derive the estimated fetal weights (EFWs) from sonographic ultrasound measurements at each of the gestational time points when the ultrasound measurements were taken and recorded. In the second step, a joint mixed-effects model with latent subject-specific random effects was fitted. One component of the joint model was a quadratic mixed-effects model to derive the predicted birth weights (PBWs). Another component was a probit mixed-effects model, from which the classification of macrosomia or LGA was determined from the PBWs by comparing the PBWs with a pre-specified threshold [6]. However, previous literature has noted that there were 26 candidate empirical formulas for estimating fetal weights [7,8,9]. In addition, some literature also argued that nonlinear mixed-effects models should be more appropriate in modeling growth curves than linear or quadratic mixed-effects models [10]. Selecting a particular EFW empirical formula and a quadratic mixed-effects model, as in Zhang et al. [4], increases the uncertainties in predicting macrosomia or LGA, because these empirical formulas and the statistical models function diversely.

In this article, we investigate the use of ensemble methods to aggregate prediction results given by different EFW empirical formulas and the statistical models. The goal of this practice is to improve the prediction in macrosomia or LGA. With ensemble methods, it is not required to select any specific statistical model or empirical formulas. Instead, the prediction capability of each combination of the empirical formulas and statistical models is combined and aggregated to generate a learning procedure that gives the best prediction performance.

## 2. Methods

### 2.1. Data

The Successive Small-for-Gestational-Age Births study (SGA study) was funded by the National Institute of Child Health and Human Development (NICHD), in the National Institutes of Health of the USA in 1983. The study was conducted concurrently by the University of Bergen in Norway, the University of Uppsala in Sweden, and the University of Alabama, USA, from 1984 to 1985 [11]. The initial goal of the SGA study was to characterize the different types of intra-uterine growth restriction and to assess the associated risk factors.

To demonstrate the strengths of ensemble methods in predicting fetal macrosomia or LGA, we took the Scandinavian data in the SGA study that were collected in Norway and Sweden. The Scandinavia SGA data were collected from January 1st_,_ 1986, through March 31st, 1988 from nulliparous (parity 1) and primiparous (parity 2) Caucasian pregnant women prior to the 20th gestational week who had a singleton pregnancy and spoke one of the Scandinavian languages. A total of 6354 women were recruited to the study, and 632 of them were excluded from the study, according to the exclusion criteria (n = 432) or due to absence of first prenatal visit (n = 200). The remaining 5722 patients were split into three subgroups. A random sample of 561 patients was first selected. Then, a “high-risk” group of 1384 patients was identified out of the random sample of 561 patients on the basis of five small-for-gestational-age (SGA) risk factors: giving birth to an infant with birthweight below 2750 g in the past, maternal cigarette smoking at conception, pre-pregnancy weight lower than 50 kg, a previous perinatal death, and the presence of chronic maternal disease (chronic renal disease, hypertension, or heart disease). The remaining 3777 patients were considered as the “low-risk” group. The patients from the random sample and “high-risk” group were eligible to participate in a detailed follow-up study, during which the women were examined at approximately 17th, 25th, 33rd, and 37th weeks of gestation. For each visit, ultrasound examination was performed, and demographic and medical information were collected. At the end of the Scandinavia SGA study, only 1945 were able to complete the follow-up study.

In our study, we restricted the study data to the 1115 women in the Scandinavia SGA study who had all four ultrasound examinations and complete covariate information (maternal age, pre-pregnancy body weight and height, previous diseases history, and smoking history) in the Scandinavia SGA study. In the ultrasound examination records in the Scandinavia SGA study, there were three fetal measurements: biparietal diameter (BPD), middle abdominal diameter (MAD), and femur length (FL).

### 2.2. An Ensemble Learning Procedure of Predicting Macrosomia and LGA

**Figure 1:**
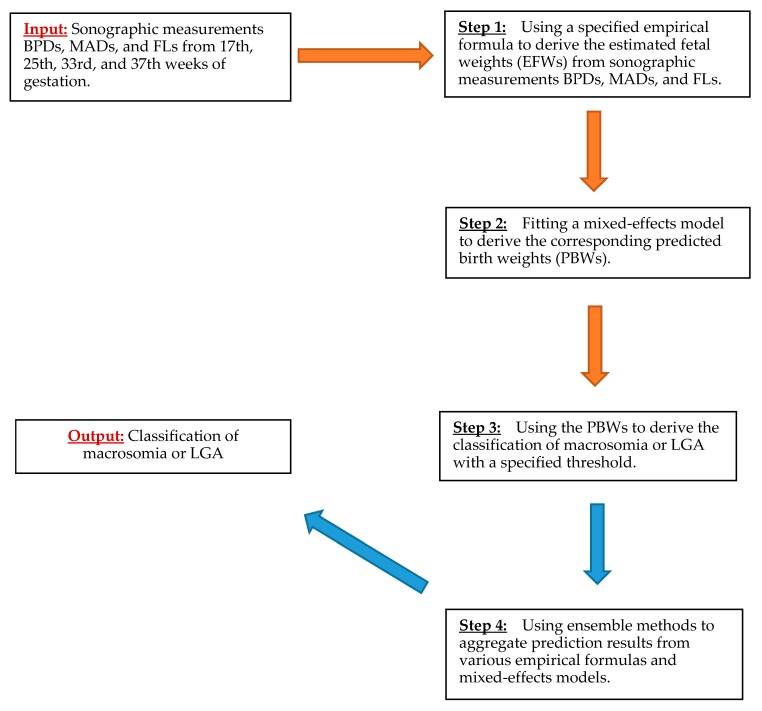
An ensemble learning procedure to predict macrosomia or LGA from prenatal ultrasound measurements.

Here, we developed a four-step ensemble learning procedure to predict macrosomia or LGA from prenatal ultrasound measurements. The output of the learning procedure is the binary classification of either macrosomia or LGA. The prediction of macrosomia and LGA is conducted based upon a set of input features. The primary input features are the sonographic measurements BPDs, MADs, and FLs collected from 17th, 25th, 33rd, and 37th weeks of gestation, as well as gestational age at delivery, in the Scandinavia SGA study. Other features also include maternal age, pre-pregnancy body mass index, parity, smoking status, existing diabetes, and gestational diabetes. Figure 1 shows a diagram that delineates the four steps in the ensemble learning procedure, to predict macrosomia or LGA. Step 1 is to take the sonographic measurements for each of the gestational weeks and each of the empirical formulas in Table A2, to obtain EFWs at each gestational time point. Step 2 is to fit either a nonlinear mixed-effects model or a quadratic mixed-effects model to predict the corresponding PBWs at birth from the EFWs obtained in Step 1. Step 3 is to use the PBWs to derive the classification of macrosomia or LGA by using a specified threshold. Step 4 is the ensemble learning step, in which prediction output from various empirical formulas and the nonlinear and quadratic mixed-effects models are combined to generate the ensemble learning prediction results.

### 2.3. Estimated Fetal Weights with 26 Empirical Formulas

In the ultrasound examination records in the Scandinavia SGA study, there were three fetal measurements BPD, MAD, and FL. Melamed et al. [7] summarized 26 different empirical sonographic formulas (see Appendix A Table A1) that can be taken to estimate fetal weights from sonographic ultrasound measures. In our ensemble learning procedure, we considered all the 26 models in our analysis. Abdominal circumference (AC) in the empirical sonographic formulas can be calculated by 3.1416 × MAD, and head circumference (HC) can be derived by the formula introduced in [12].

### 2.4. Mixed-Effects Models for Predicting PBWs and Deriving the Classification of Macrosomia or LGA

We built a nonlinear three-parameter mixed-effects logistic model with latent random effects to predict PBWs and derive the classification of macrosomia or LGA [10,13]. The three-parameter mixed-effects logistic model for the ith fetus (i=1,⋯,n indexing the study subjects) at gestational time $t_{ij}$ (j=1, 2, 3, 4 indexing the time when the gestational ultrasound measurements were recorded, j=5 indexing the time of birth) is as follows:

$$y_{ij}=\frac{\phi_{1i}}{1+\exp\left[-\left(t_{ij}-\phi_{2i}\right)/\phi_{3i}\right]}+\epsilon_{ij},$$

where $y_{ij}$ is the EFW obtained from one of the 26 formulas in Table A1 at gestational time $t_{ij}$, j=1, 2, 3, and 4, and $y_{ij}$ is the birth weight when j=5, $\phi_{i}=\left(\phi_{1i},\;\phi_{2i},\;\phi_{3i}\right)$ are model parameters in which $\phi_{1i}$ indicates amplitude, $\phi_{2i}$ indicates the smoothness, $\phi_{3i}$ indicates stretch, and $\epsilon_{ij}$ are within-subject random errors. For the nonlinear mixed-effects model, each parameter $\phi_{ki}$ (k=1, 2, 3 indexing the parameter), can be further modeled by a linear representation:

$$\phi_{ki}=X_{ij}^{\prime }\beta_{k}+b_{ki},$$

where $X_{ij}$ is a vector of fixed-effects the covariates of maternal age, pre-pregnancy body mass index, previous disease history, including diabetes, cardiac disease, high blood pressure, renal disorders, and other diseases, and smoking during pregnancy; and $\beta_{k}$ denotes the corresponding regression parameters. The first element in $X_{ij}$ equals 1 for the interception. In our learning procedure, the covariates were included in the linear term $\phi_{1i}=X_{ij}^{\prime }\beta_{1}+b_{1i}$, and $\phi_{2i}$ and $\phi_{3i}$ were specified as $\phi_{2i}=\beta_{2}+b_{2i}$ and $\phi_{3i}=\beta_{3}+b_{3i}$. We also considered the nonlinear three-parameter mixed-effects logistic model without any covariates in $\phi_{1i}$ such that $\phi_{1i}=\beta_{1}+b_{1i}$. The random effects $b_{i}=\left(b_{1i},\;b_{2i},b_{3i}\right)\prime$ represent the latent individual variations that are not explained by the covariates. We assumed the random effects, $b_{i}$, independently follow a multivariate normal distribution, with a vector of mean 0 and a variance-covariance matrix Σ, where Σ was assumed to be positive, definite, and unstructured. Further, we assumed the following heteroscedastic model [10] for the within-subject random errors $\epsilon_{ij}$:

$$\epsilon_{ij}∼N\left(0,\;{\left|\frac{\phi_{1i}}{1+\exp\left[-\left(t_{ij}-\phi_{2i}\right)/\phi_{3i}\right]}\right|}^{2\delta }\right).$$

We compared the above nonlinear mixed-effects models with the following quadratic mixed-effects model implemented in Zhang et al. [4].

$$y_{ij}=X\prime_{ij}\beta +\theta_{1}t_{ij}+\theta_{2}t_{ij}^{2}+b_{i0}+b_{i1}t_{ij}+b_{i2}t_{ij}^{2}+\epsilon_{ij}.$$

The configuration of $X_{ij}$, β, $b_{i},$ and $\epsilon_{ij}$ is identical to those in the nonlinear mixed-effects model, and $\theta =\left(\theta_{1},\;\theta_{2}\right)$ are the parameters of time fixed-effects $t_{ij}$ and $t_{ij}^{2}$. For the quadratic mixed-effects model, we also considered the one without any covariates for predicting the birth weights and deriving the classification of macrosomia or LGA.

### 2.5. Ensemble Learning Methods

Here, we propose to apply ensemble methods [14], to combine the prediction results generated from 26 individual EFW empirical formulas and from nonlinear and linear mixed-effects models. One appealing property for ensemble methods is that they combine the classification strengths of individual models but do not overfit the data. Two types of ensemble algorithms, majority voting and stacking, are considered. Majority voting is one of the most fundamental ensemble methods for classification [14]. For a binary classification problem, the final classification of majority voting is the class that receives more than half of the votes from the individual learning models. Because the individual learning models can be correlated, it is necessary to select among individual learning models that are combined in majority voting [15,16,17]. Here, we implemented least absolute shrinkage and selection operator (LASSO) [18], smoothed clipped absolute deviation (SCAD) [19], and minimax penalized likelihood (MCP) [20] to select individual learning models. 

The stacking method [21,22] is one of the most known meta-learning methods. It combines the prediction results from several different individual learning models, called “first-level learners”, by another learning model, named “second-level learner” or “meta-learner”. Van der Laan et al. [23] proposed to train the meta-learner by a unified cross-validation algorithm or super learner and proved its oracle properties. The unified cross-validation algorithm is summarized as follows. For a K-fold cross-validation procedure (K=10 is set here), the training dataset was split into K equal-sized groups, stratified by the response variable. Then, let the k-th group be the validation data, take the remaining data to train the first-level learners, and collect the prediction values from the validation data as the covariates of the meta-leaner. By repeating the above procedure on every fold of data, along with the original response variable, a complete dataset, called the “leave-one data”, is generated for training the meta-learner. Several reports [21,22,24] proposed using the logistic regression with positive constraint on the regression coefficients as the meta-learner for classification, whereas Van der Laan et al. [23] used linear regression as the meta-learner for regression models. Debray et al. [24] suggested performing model selection on the meta-learner, so we applied LASSO, SCAD, and MCP to conduct model selection on the meta-learner.

### 2.6. Evaluation of Prediction Performance

In our investigation, the original dataset was randomly divided into a training dataset (70%, n = 781) and a testing dataset (30%, n = 334), stratified by the presence of macrosomia or LGA. For an individual mixed-effects model with a particular EFW empirical formula, the entire training dataset, along with the ultrasound measures and demographic information in the testing dataset, was used to fit the model. The birth time, birthweight, and the true macrosomia or LGA status in the testing dataset were used to evaluate the prediction performance. For ensemble methods, the ensemble learner was trained by the training dataset and tested by the testing dataset.

The prediction accuracy of each individual mixed-effects model with a particular EFW empirical formula, as well as the ensemble learner, was assessed by the areas under the receiver operating characteristic curve (AUC), sensitivity, specificity, positive predictive value (+PV), negative predictive value (−PV), positive likelihood ratio (+LR), negative likelihood ratio (−LR), and Youden’s index (sensitivity + specificity − 1) for predicting both macrosomia and LGA.

## 3. Results

Table 1 shows the baseline characteristics of our study subjects (n = 1115). The mean maternal age of the study subjects was 28 years (standard deviation or SD: 4 years), and the average height and weight before pregnancy were 166 centimeters (SD: 6 centimeters) and 59 kg (SD: 10 kg), respectively. Twenty-one percent of women had a history of SGA births, and about half of them smoked at enrollment. Only a few had high blood pressure, cardiac disease, diabetes, or renal disorder, but 15% had other types of diseases. The mean gestational age at birth was 280 days (SD: 8 days), and the mean birthweight was 3562 g (SD: 478 g). Seventeen percent of infants had macrosomia, 11% had LGA at birth, and, among the LGA infants, 9 of them (7%) were not macrosomia infants. The current study sample was used as the reference of LGA [11].

### 3.1. Prediction Performance of Individual Models and Empirical Formulas in Macrosomia

We predicted macrosomia or LGA with the nonlinear and quadratic mixed-effects models described above, and also considered those models with or without the covariates. Thus, four mixed-effects models combined with 26 empirical formulas for EFWs, totally 104 learning models, were fitted. The prediction performance of individual learning models in predicting macrosomia is reported in Appendix A Table A2, Table A3, Table A4 and Table A5. For the three-parameter mixed-effects logistic models, their prediction performance varied with different EFW empirical formulas, and adding covariates into the models did not improve their prediction performance (see Appendix A Table A2 and Table A3). The three-parameter mixed-effects logistic models, either with or without covariates, predicted all birth weights to be under 4000 g when combined with empirical formula 3 in Table A1. The two nonlinear mixed-effects models combined with empirical formula 11 in Table A1 gave the highest value of Youden’s index of 0.670, and the lowest value of Youden’s index of 0.050 was obtained by empirical formula 18 in Table A1 without covariates. The best sensitivities of 0.857 and 0.839 were obtained from the two models with or without covariates, respectively, when coupled with empirical formula 7 in Table A1. The AUCs for these models ranged from 0.871 to 0.910. The prediction performance of the quadratic mixed-effects models also varied among different empirical formulas, and adding covariates did not improve their prediction performance either (see Appendix A Table A4 and Table A5). The quadratic mixed-effects models combined with empirical formula 12 in Table A1 gave the highest value of Youden’s index of 0.663, whereas the lowest value of Youden’s index of 0.310 was obtained by empirical formula 3, in Table A1, without covariates. The best sensitivity of 0.982 was obtained from the two models with or without covariates when coupled with empirical formulas 7 and 11 in Table A1. The AUCs for these models ranged from 0.857 to 0.905.

### 3.2. Prediction Performance of Individual Models and Empirical Formulas in LGA

LGA is defined as a newborn with a birthweight greater than the 90th percentile for gestational age. Here, given the input of sonographic ultrasound measures, both the nonlinear and quadratic mixed-effects models can generate PBWs for each of fetuses at any time point, regardless of its actual birth time. A newborn was classified as LGA if his or her PBW was above the 90th percentile of PBWs of all other fetuses, when the fetuses were assumed to be born at the identical birth time as that newborn. The prediction performance of the nonlinear and quadratic mixed-effects models is reported in Appendix A Table A6, Table A7, Table A8 and Table A9. The prediction performance showed small variation among different EFWs empirical formulas and among these models. The Youden’s indexes ranged from 0.354 to 0.526, the sensitivities ranged from 0.424 to 0.576, the specificities ranged from 0.930 to 0.950, and the AUCs ranged from 0.863 to 0.894.

### 3.3. Prediction Performance of Ensemble Methods

Two ensemble methods, voting and stacking methods, were applied to combine the 104 learning models for the prediction of macrosomia. The voting method was implemented by using two approaches: voting from all learning models without selection and voting from the selected learning models. To select among the learning models, a penalized logistic regression was run with either a LASSO, SCAD, or MCP penalty. When the stacking method was implemented, we fit a linear regression model as our meta-learner, either without variable selection or with three variable selection methods, LASSO, SCAD, and MCP. The prediction performance of the ensemble methods for the prediction of macrosomia is summarized in Table 2. The voting and stacking methods with the SCAD selection yielded the best Youden’s indexes of 0.681 and 0.688, respectively, which were higher than the Youden’s indexes generated by any other individual learning models in Table A2, Table A3, Table A4 and Table A5. The voting method with the SCAD and MCP selection and the stacking method with the LASSO, SCAD, and MCP selection outperformed most of the individual learning models listed in Table A2, Table A3, Table A4 and Table A5. The voting method with the SCAD or MCP model selection generated an AUC of 0.932 and 0.924, respectively, higher than the AUCs generated by any other individual learning models in Table A2, Table A3, Table A4 and Table A5.

We also applied the voting and stacking methods for the prediction of LGA, to combine the 104 learning models. The voting method was implemented as described in the prediction of macrosomia. When the stacking method was implemented, we fit a logistic regression with positive constraints [22], without further selection on first-level learners, and a logistic regression with the LASSO, SCAD, and MCP selection on first-level learners as our meta-learner. The results are summarized in Table 3. These results showed that the best prediction results were supplied by the voting method with the MCP selection with a Youden’s index of 0.537 and a sensitivity of 0.636, both higher than any of the individual learning models in Table A6, Table A7, Table A8 and Table A9.

## 4. Discussion and Conclusions

We proposed using ensemble methods to combine the strengths from nonlinear and quadratic mixed-effects models and 26 empirical formulas of EFWs to predict macrosomia and LGA of newborns from sonographic ultrasound measurements. The prediction performance of the ensemble methods was studied with the data from the Scandinavia SGA study. We showed that the prediction performance varied among the empirical formulas and mixed-effects models. The three-parameter mixed-effects logistic model combined with empirical formula 11 in Table A1 gave the best prediction results in predicting macrosomia. In predicting LGA, the prediction performance also varied. The best prediction results were obtained from the quadratic mixed-effects model combined with empirical formula 6 in Table A1. These results showed that it was difficult to select any individual statistical learning model combined with only one empirical formula of EFWs, to predict macrosomia or LGA from sonographic ultrasound measurements.

We subsequently proposed applying ensemble methods to aggregate the prediction results from mixed-effects models and empirical formulas of EFWs, to achieve better prediction performance. Our investigation showed that, with the aid of either the SCAD or MCP for model selection, both stacking and voting methods improved the prediction accuracies in predicting macrosomia, as opposed to those from individual statistical models and empirical formulas. The voting method with the MCP for model selection predicted LGA more accurately than the individual statistical models and empirical formulas. In this study, the ensemble prediction algorithms were created from all the prenatal ultrasound measures and birth weights. However, the algorithms can be used to make prediction on macrosomia or LGA with the ultrasound measures only from the first or second trimester, although the ultrasound measures collected in the third trimester or before birth can substantially improve the prediction accuracy. Our current study is a feasibility study that demonstrates the ensemble methods can be integrated with ultrasound examinations to assist obstetricians in clinical diagnosis on whether a pregnant woman will give birth to a large infant, and to further guide clinical interventions for the condition. However, measurable clinical benefits are unclear until they are demonstrated in prospective clinical studies.

Our current study has several limitations. The proportion of macrosomia or LGA infants in the Scandinavia SGA dataset is small (15% for macrosomia and 11% LGA of n = 1115 infants). This may largely influence the prediction accuracy given by the individual models and empirical formulas. However, using machine learning methods specifically ensemble methods, can accommodate such imbalanced data, and improve prediction accuracy [23]. In addition, the initial objective of the SGA study was to characterize the intra-uterine growth restriction and assess the associated risk factors of SGA, but the study was not designed for studying macrosomia or LGA. As a consequence, the risk factors associated with macrosomia or LGA were not thoroughly collected. This may be the reason why, in our study, we did not receive benefits from adding covariates into the mixed-effects models. Lastly, The SGA study was conducted in 1980s, with out-of-date sonographic ultrasound examination technologies, so the prediction models developed in this study may not be directly applied to predict macrosomia or LGA with the ultrasound measures from most recent state-of-the-art ultrasound technologies. However, the ensemble methods can still be applied to aggregate any available ultrasound prediction models. Also, the subjects in this study were the Caucasians from Europe that were mostly not obese (11.6% overweight rate and 2.1% obese rate in the study). New studies and data on a diverse population should be able to substantially improve the prediction of macrosomia and LGA among both the whites and minorities, as well as the overweight and obese population.

## Figures and Tables

**Table 1:** Baseline characteristics of study subjects.

Demographic Characteristic	Mean (Standard Deviation) n = 1115
Maternal age (years)	28.32 (4.12)
Birthweight (grams)	3562 (478)
Gestational age (days)	279.99 (8.34)
Maternal height (centimeters)	165.96 (5.99)
Maternal weight (kilograms)	59.20 (10.00)
**Health History**	**Number of Subjects (Percentage)**
High blood pressure	20 (1.8)
Cardiac disease	10 (0.9)
Diabetes	3 (0.3)
Renal disorders	11 (1.0)
Other diseases	172 (15.4)
Small for gestational age	235 (21.1)
Large for gestational age	124 (11.1)
Macrosomia	195 (17.5)
Smoking at enrollment (cigarettes/day)	
0	436 (49.1)
1–9	180 (16.1)
10–19	397 (35.6)
20+	102 (9.2)

**Table 2:** Prediction performance of macrosomia by the ensemble methods.

Method	AUC (95% CI)	Sensitivity (95% CI)	Specificity (95% CI)	+LR (95% CI)	−LR (95% CI)	+PV (95% CI)	−PV (95% CI)	Youden’s Index
Stacking: no selection	0.852 (0.805, 0.898)	0.607 (0.468, 0.735)	0.867 (0.821, 0.905)	4.562 (3.162, 6.582)	0.453 (0.326, 0.630)	0.479 (0.359, 0.601)	0.916 (0.876, 0.900)	0.474
Stacking: LASSO	0.907 (0.891, 0.943)	0.768 (0.636, 0.870)	0.874 (0.829, 0.911)	6.099 (4.334, 8.582)	0.266 (0.165, 0.429)	0.551 (0.434, 0.664)	0.949 (0.915, 0.973)	0.642
Stacking: SCAD	0.909 (0.873, 0.944)	0.839 (0.717, 0.924)	0.849 (0.801, 0.889)	5.555 (4.110, 7.509)	0.189 (0.104, 0.345)	0.528 (0.419, 0.635)	0.963 (0.931, 0.983)	0.688
Stacking: MCP	0.907 (0.872, 0.943)	0.786 (0.656, 0.844)	0.863 (0.817, 0.901)	5.748 (4.151, 7.960)	0.258 (0.150, 0.411)	0.537 (0.423, 0.647)	0.952 (0.918, 0.975)	0.649
Voting: no selection	0.903 (0.887, 0.919)	0.613 (0.553, 0.670)	0.912 (0.900, 0.927)	6.991 (5.726, 8.536)	0.425 (0.366, 0.492)	0.608 (0.549, 0.665)	0.914 (0.897, 0.929)	0.525
Voting: LASSO	0.891 (0.871, 0.911)	0.542 (0.482, 0.601)	0.959 (0.947, 0.969)	8.885 (6.988, 11.296)	0.809 (0.552, 0.673)	0.664 (0.599, 0.724)	0.902 (0.885, 0.918)	0.481
Voting: SCAD	0.932 (0.918, 0.945)	0.845 (0.798, 0.885)	0.836 (0.814, 0.855)	5.143 (4.501, 5.876)	0.185 (0.141, 0.244)	0.681 (0.612, 0.741)	0.533 (0.486, 0.580)	0.681
Voting: MCP	0.924 (0.910, 0.938)	0.810 (0.759, 0.854)	0.854 (0.834, 0.873)	5.565 (4.817, 6.428)	0.223 (0.175, 0.283)	0.553 (0.504, 0.601)	0.953 (0.939, 0.964)	0.664

Areas under the receiver operating characteristic curve (AUC), confident interval (CI), positive likelihood ratio (+LR), negative likelihood ratios (−LR), positive predictive values (+PV), negative predictive values (−PV).

**Table 3:** Prediction performance of large for gestational age by the ensemble methods.

Method	AUC (95% CI)	Sensitivity (95% CI)	Specificity (95% CI)	+LR (95% CI)	−LR (95% CI)	+PV (95% CI)	−PV (95% CI)	Youden’s Index
Stacking: no selection	0.785 (0.697, 0.874)	0.485 (0.308, 0.665)	0.947 (0.915, 0.969)	9.121 (5.044, 16.495)	0.544 (0.390, 0.758)	0.500 (0.319, 0.681)	0.944 (0.911, 0.967)	0.432
Stacking: LASSO	0.811 (0.728, 0.894)	0.455 (0.281, 0.636)	0.944 (0.911, 0.967)	8.048 (4.443, 14.578)	0.578 (0.423, 0.790)	0.469 (0.291, 0.653)	0.940 (0.907, 0.964)	0.398
Stacking: SCAD	0.785 (0.696, 0.873)	0.455 (0.281, 0.636)	0.944 (0.911, 0.967)	8.048 (4.443, 14.578)	0.578 (0.423, 0.790)	0.469 (0.291, 0.653)	0.940 (0.907, 0.964)	0.398
Stacking: MCP	0.775 (0.688, 0.863)	0.485 (0.308, 0.665)	0.937 (0.903, 0.962)	7.681 (4.389, 13.441)	0.550 (0.394, 0.767)	0.457 (0.288, 0.634)	0.943 (0.911, 0.967)	0.421
Voting: no selection	0.823 (0.740, 0.906)	0.485 (0.308, 0.665)	0.950 (0.919, 0.972)	9.729 (5.309, 17,831)	0.542 (0.389, 0.756)	0.516 (0.331, 0.698)	0.944 (0.912, 0.967)	0.435
Voting: LASSO	0.811 (0.728, 0.895)	0.515 (0.335, 0.692)	0.940 (0.907, 0.964)	8.614 (4.936, 15.035)	0.516 (0.362, 0.734)	0.486 (0.314, 0.660)	0.946 (0.915, 0.969)	0.455
Voting: SCAD	0.799 (0.713, 0.885)	0.515 (0.335, 0.692)	0.944 (0.911, 0.967)	9.121 (5.168, 16.099)	0.514 (0.361, 0.731)	0.500 (0.319, 0.681)	0.947 (0.915, 0.969)	0.459
Voting: MCP	0.774 (0.687, 0.861)	0.636 (0.451, 0.796)	0.900 (0.861, 0.932)	6.385 (4.168, 9.780)	0.404 (0.257, 0.635)	0.412 (0.276, 0.558)	0.958 (0.927, 0.978)	0.537

Areas under the receiver operating characteristic curve (AUC), confident interval (CI), positive likelihood ratio (+LR), negative likely ratios (−LR), positive predictive values (+PV), negative predictive values (−PV).

## Appendix A

**Table A1:** Empirical formulas used for estimating fetal weight from sonographic ultrasound measurements (Melamed et al. [5]).

*Formula*	Reference	Equation
1	Campbell and Wilkin [25]	$\ln EFW=-4.564+0.383\cdot AC-0.00331\cdot AC^{2}$
2	Hadlock et al. [5]	$\ln EFW=2.695+0.253\cdot AC-0.00275\cdot AC^{2}$
3	Jordaan [26]	$\log_{10}EFW=0.6328+0.1881\cdot AC-0.0043\cdot AC^{2}+0.000036239\cdot AC^{3}$
4	Warsof et al. [27]	$\log_{10}EFW=1.1633+0.092\cdot AC-0.000019\cdot AC$
5	Higginbottom et al. [28]	$EFW=0.0816\cdot AC^{3}$
6	Hadlock et al. [5]	$\log_{10}EFW=1.304+0.05281\cdot AC+0.1938\cdot FL-0.004\cdot AC\cdot FL$
7	Woo et al. [29]	$\log_{10}EFW=0.59+0.08\cdot AC+0.28\cdot FL-\;0.00716\cdot AC\cdot FL$
8	Warsof et al. [30]	$\ln EFW=2.792+0.108\cdot FL+0.0036\cdot AC^{2}-0.0027\cdot AC\cdot FL$
9	Vintzileos et al. [31]	$\log_{10}EFW=1.879+0.084\cdot BPD+0.026\cdot AC$
10	Warsof et al. [27]	$\log_{10}EFW=1.401+0.144\cdot BPD+0.032\cdot AC-0.000111\cdot AC\cdot BPD^{2}$
11	Shepard et al. [32]	$\log_{10}EFW=1.2508+0.166\cdot BPD+0.046\cdot AC-0.002546\cdot BPD\cdot AC$
12	Jordaan [26]	$\log_{10}EFW=1.9317+0.0377\cdot AC+0.0950\cdot BPD-0.0015\cdot BPD\cdot AC$
13	Hadlock et al. [5]	$\log_{10}EFW=1.1134+0.05845\cdot AC-0.000604\cdot AC^{2}-0.007365\cdot BPD^{2}+0.000595\cdot BPD\cdot AC+0.1694\cdot BPD$
14	Woo et al. [29]	$\log_{10}EFW=1.63+0.16\cdot BPD+0.00111\cdot AC^{2}-0.0000859\cdot BPD\cdot AC^{2}$
15	Mirghani et al. [33]	$\log_{10}EFW=2.1315+0.0056541\cdot AC\cdot BPD-0.00015515\cdot BPD\cdot AC^{2}+0.000019782\cdot AC^{3}+0.052594\cdot BPD$
16	Hadlock et al. [5]	$\log_{10}EFW=1.182+0.0273\cdot HC+0.07057\cdot AC-0.00063\cdot AC^{2}-0.0002184\cdot HC\cdot AC$
17	Jordaan [26]	$\log_{10}EFW=0.9119+0.0488\cdot HC+0.0824\cdot AC-0.001599\cdot HC\cdot AC$
18	Jordaan [26]	$\log_{10}EFW=2.3231+0.02904\cdot AC+0.0079\cdot HC-0.0058\cdot BPD$
19	Hadlock et al. [5]	$\log_{10}EFW=1.335-0.0034\cdot AC\cdot FL+0.0316\cdot BPD+0.0457\cdot AC+0.1623\cdot FL$
20	Woo et al. [29]	$\log_{10}EFW=1.54+0.15\cdot BPD+0.00111\cdot AC^{2}-0.0000764\cdot BPD\cdot AC^{2}+0.05\cdot FL-0.000992\cdot FL\cdot AC$
21	Shinozuka et al. [34]	$EFW=0.23966\cdot AC^{2}\cdot FL+1.6230\cdot BPD^{3}$
22	Mirghani et al. [33]	$\log_{10}EFW=2.7193+0.0094962\cdot AC\cdot BPD-0.1432\cdot FL-0.00076742\cdot AC\cdot BPD^{2}+0.001745\cdot FL\cdot BPD^{2}$
23	Hadlock et al. [5]	$\log_{10}EFW=1.326-0.00326\cdot AC\cdot FL+0.0107\cdot HC+0.0438\cdot AC+0.158\cdot FL$
24	Combs et al. [35]	$EFW=0.23718\cdot AC^{2}\cdot FL+0.03312\cdot HC^{3}$
25	Ott et al. [36]	$\log_{10}EFW=0.9339+0.04355\cdot HC+0.05392\cdot AC-0.0008582\cdot HC\cdot AC+1.2594\cdot \left(FL/AC\right)$
26	Hadlock et al. [5]	$\log_{10}EFW=1.3596+0.0064\cdot HC+0.0424\cdot AC+0.174\cdot FL+0.00061\cdot BPD\cdot AC-0.00386\cdot AC\cdot FL$

Abdominal circumference (AC), femur length (FL), biparietal diameter (BPD), and head circumference (HC) are expressed in centimeters, and EFW is expressed in grams, unless stated otherwise. This is an identical table to the Table 1 in Melamed et al. [5].

**Table A2:** Prediction performance of the three-parameter mixed-effects logistic model without covariance when combined with the 26 estimated fetal weight empirical formulas * in predicting macrosomia.

*Formulas*	AUC (95% CI)	Sensitivity (95% CI)	Specificity (95% CI)	+LR (95% CI)	−LR (95% CI)	+PV (95% CI)	−PV (95% CI)	Youden’s Index
1	0.884 (0.839, 0.930)	0.250 (0.144, 0.384)	0.982 (0.958, 0.994)	13.900 (5.217, 37.033)	0.764 (0.656, 0.889)	0.737 (0.488, 0.910)	0.867 (0.824, 0.902)	0.232
2	0.885 (0.840, 0.930)	0.464 (0.330, 0.603)	0.946 (0.913, 0.969)	8.605 (4.881, 15.169)	0.566 (0.443, 0.724)	0.634 (0.469, 0.779)	0.898 (0.857, 0.930)	0.410
3	All predicted birth weights were under 4000 g.							
4	0.886 (0.842, 0.931)	0.571 (0.432, 0.703)	0.914 (0.874, 0.944)	6.619 (4.243, 10.325)	0.469 (0.346, 0.636)	0.571 (0.432, 0.703)	0.914 (0.874, 0.944)	0.485
5	0.883 (0.838, 0.928)	0.536 (0.397, 0.670)	0.932 (0.895, 0.958)	7.838 (4.765, 12.895)	0.498 (0.375, 0.661)	0.612 (0.462, 0.748)	0.909 (0.869, 0.940)	0.467
6	0.882 (0.835, 0.928)	0.464 (0.330, 0.603)	0.924 (0.887, 0.953)	6.146 (3.734, 10.116)	0.579 (0.453, 0.741)	0.553 (0.401, 0.698)	0.895 (0.854, 0.928)	0.389
7	0.882 (0.837, 0.926)	0.857 (0.738, 0.936)	0.799 (0.747, 0.844)	4.255 (3.290, 5.504)	0.179 (0.094, 0.341)	0.462 (0.363, 0.562)	0.965 (0.933, 0.985)	0.656
8	0.872 (0.824, 0.920)	0.429 (0.297, 0.568)	0.932 (0.895, 0.958)	6.271 (3.695, 10.643)	0.613 (0.488, 0.771)	0.558 (0.399, 0.709)	0.890 (0.848, 0.924)	0.360
9	0.907 (0.871, 0.942)	0.821 (0.696, 0.911)	0.845 (0.797, 0.886)	5.311 (3.931, 7.174)	0.211 (0.120, 0.371)	0.517 (0.408, 0.624)	0.959 (0.926, 0.980)	0.667
10	0.906 (0.867, 0.945)	0.536 (0.397, 0.670)	0.953 (0.921, 0.975)	11.456 (6.388, 20.544)	0.487 (0.367, 0.646)	0.698 (0.539, 0.828)	0.911 (0.872, 0.941)	0.489
11	0.904 (0.867, 0.942)	0.821 (0.696, 0.911)	0.849 (0.801, 0.889)	5.437 (4.011, 7.370)	0.210 (0.120, 0.370)	0.523 (0.414, 0.630)	0.959 (0.927, 0.980)	0.670
12	0.910 (0.873, 0.946)	0.643 (0.504, 0.766)	0.921 (0.883, 0.950)	8.123 (5.201, 12.689)	0.388 (0.272, 0.552)	0.621 (0.484, 0.745)	0.928 (0.890, 0.955)	0.564
13	0.908 (0.869, 0.946)	0.696 (0.559, 0.812)	0.910 (0.870, 0.941)	7.744 (5.129, 11.693)	0.334 (0.224, 0.497)	0.609 (0.479, 0.729)	0.937 (0.901, 0.963)	0.607
14	0.908 (0.870, 0.946)	0.214 (0.116, 0.344)	0.989 (0.969, 0.998)	19.857 (5.792, 68.082)	0.792 (0.692, 0.911)	0.800 (0.519, 0.898)	0.862 (0.819, 0.898)	0.203
15	0.910 (0.874, 0.947)	0.732 (0.597, 0.842)	0.910 (0.870, 0.941)	9.141 (5.424, 12.220)	0.294 (0.191, 0.455)	0.621 (0.493, 0.738)	0.944 (0.909, 0.968)	0.642
16	0.905 (0.865, 0.969)	0.429 (0.297, 0.568)	0.968 (0.939, 0.985)	13.238 (6.507, 26.933)	0.591 (0.470, 0.742)	0.727 (0.545, 0.867)	0.894 (0.853, 0.926)	0.396
17	0.871 (0.824, 0.917)	0.339 (0.218, 0.478)	0.946 (0.913, 0.969)	6.288 (3.406, 11.608)	0.698 (0.578, 0.844)	0.559 (0.379, 0.728)	0.877 (0.834, 0.912)	0.285
18	0.888 (0.848, 0.928)	0.054 (0.011, 0.149)	0.996 (0.980, 1.000)	14.893 (1.578, 140.579)	0.950 (0.892, 1.011)	0.750 (0.194, 0.994)	0.839 (0.795, 0.877)	0.050
19	0.901 (0.860, 0.943)	0.679 (0.540, 0.797)	0.924 (0.887, 0.953)	8.983 (5.734, 12.074)	0.348 (0.237, 0.509)	0.644 (0.509, 0.764)	0.935 (0.899, 0.961)	0.603
20	0.910 (0.871, 0.948)	0.607 (0.468, 0.735)	0.935 (0.900, 0.961)	9.377 (5.722, 15.367)	0.420 (0.303, 0.583)	0.654 (0.509, 0.780)	0.922 (0.884, 0.950)	0.542
21	0.905 (0.865, 0.945)	0.429 (0.297, 0.568)	0.950 (0.917, 0.972)	8.510 (4.702, 15.403)	0.602 (0.479, 0.756)	0.632 (0.460, 0.782)	0.892 (0.851, 0.925)	0.378
22	0.901 (0.865, 0.937)	0.179 (0.089, 0.304)	0.982 (0.959, 0.994)	9.929 (3.529, 27.934)	0.836 (0.740, 0.946)	0.667 (0.384, 0.882)	0.856 (0.812, 0.892)	0.161
23	0.902 (0.861, 0.943)	0.464 (0.330, 0.603)	0.939 (0.904, 0.964)	7.592 (4.426, 13.025)	0.571 (0.446, 0.730)	0.605 (0.444, 0.750)	0.897 (0.856, 0.929)	0.403
24	0.906 (0.886, 0.945)	0.232 (0.130, 0.364)	0.993 (0.974, 0.999)	32.268 (7.488, 133.049)	0.773 (0.669, 0.894)	0.867 (0.595, 0.983)	0.865 (0.823, 0.901)	0.225
25	0.906 (0.866, 0.946)	0.393 (0.265, 0.532)	0.960 (0.930, 0.980)	9.929 (5.109, 19.294)	0.632 (0.511, 0.781)	0.667 (0.482, 0.820)	0.887 (0.846, 0.920)	0.353
26	0.903 (0.863, 0.944)	0.661 (0.522, 0.782)	0.932 (0.895, 0.958)	9.667 (6.025, 15.512)	0.364 (0.252, 0.526)	0.661 (0.522, 0.782)	0.932 (0.895, 0.958)	0.592

***** See Table A1 for specifications of empirical formulas. Areas under the receiver operating characteristic curve (AUC), confident interval (CI), positive likelihood ratio (+LR), negative likelihood ratios (−LR), positive predictive values (+PV), negative predictive values (−PV).

**Table A3:** Prediction performance of the three-parameter mixed-effects logistic model with covariance when combined with the 26 estimated fetal weight empirical formulas* in predicting macrosomia.

*Formulas*	AUC (95% CI)	Sensitivity (95% CI)	Specificity (95% CI)	+LR (95% CI)	−LR (95% CI)	+PV (95% CI)	−PV (95% CI)	Youden’s Index
1	0.885 (0.840, 0.931)	0.232 (0.130, 0.364)	0.982 (0.958, 0.994)	12.907 (4.793, 34.758)	0.782 (0.676, 0.904)	0.722 (0.465, 0.903)	0.864 (0.821, 0.900)	0.215
2	0.885 (0.830, 0.930)	0.500 (0.363, 0.637)	0.942 (0.908, 0.967)	8.688 (5.047, 14.953)	0.531 (0.408, 0.690)	0.636 (0.478, 0.776)	0.903 (0.863, 0.935)	0.442
3	All predicted birth weights were under 4000 g.							
4	0.885 (0.840, 0.930)	0.607 (0.468, 0.735)	0.917 (0.878, 0.947)	7.339 (4.705, 11.446)	0.428 (0.309, 0.594)	0.596 (0.458, 0.724)	0.921 (0.882, 0.950)	0.524
5	0.883 (0.837, 0.928)	0.518 (0.380, 0.653)	0.931 (0.895, 0.958)	7.577 (4.586, 12.520)	0.518 (0.394, 0.680)	0.604 (0.453, 0.742)	0.906 (0.866, 0.937)	0.450
6	0.880 (0.834, 0.927)	0.464 (0.330, 0.603)	0.928 (0.891, 0.956)	6.454 (3.886, 10.717)	0.577 (0.451, 0.738)	0.565 (0.411, 0.711)	0.896 (0.855, 0.929)	0.392
7	0.882 (0.838, 0.926)	0.839 (0.717, 0.924)	0.799 (0.747, 0.844)	4.166 (3.211, 5.407)	0.201 (0.110, 0.367)	0.456 (0.358, 0.557)	0.961 (0.927, 0.982)	0.638
8	0.880 (0.834, 0.927)	0.464 (0.330, 0.603)	0.928 (0.891, 0.956)	6.454 (3.886, 10.717)	0.577 (0.451, 0.738)	0.565 (0.411, 0.711)	0.896 (0.855, 0.929)	0.392
9	0.906 (0.870, 0.942)	0.804 (0.676, 0.898)	0.845 (0.797, 0.886)	5.195 (3.534, 7.039)	0.232 (0.136, 0.396)	0.511 (0.402, 0.619)	0.955 (0.921, 0.977)	0.649
10	0.907 (0.867, 0.946)	0.500 (0.363, 0.637)	0.950 (0.917, 0.972)	9.929 (5.594, 17.622)	0.527 (0.405, 0.685)	0.667 (0.505, 0.804)	0.904 (0.864, 0.935)	0.500
11	0.904 (0.867, 0.942)	0.821 (0.696, 0.911)	0.849 (0.801, 0.889)	5.437 (4.011, 7.370)	0.210 (0.120, 0.370)	0.523 (0.414, 0.630)	0.959 (0.926, 0.980)	0.670
12	0.910 (0.873, 0.946)	0.643 (0.504, 0.766)	0.921 (0.553, 0.950)	8.123 (5.201, 12.689)	0.388 (0.272, 0.552)	0.621 (0.484, 0.745)	0.928 (0.890, 0.955)	0.564
13	0.907 (0.868, 0.945)	0.679 (0.540, 0.797)	0.914 (0.874, 0.944)	7.860 (5.150, 11.996)	0.352 (0.240, 0.516)	0.613 (0.481, 0.734)	0.934 (0.897, 0.960)	0.592
14	0.909 (0.871, 0.947)	0.214 (0.116, 0.344)	0.986 (0.964, 0.996)	14.893 (4.984, 44.498)	0.797 (0.695, 0.915)	0.750 (0.476, 0.927)	0.862 (0.819, 0.898)	0.200
15	0.910 (0.874, 0.947)	0.714 (0.578, 0.827)	0.910 (0.870, 0.941)	7.943 (5.277, 11.956)	0.314 (0.207, 0.476)	0.615 (0.486, 0.733)	0.941 (0.905, 0.966)	0.624
16	0.905 (0.865, 0.944)	0.411 (0.281, 0.550)	0.968 (0.939, 0.985)	12.687 (6.205, 25.937)	0.609 (0.489, 0.759)	0.719 (0.533, 0.863)	0.891 (0.850, 0.924)	0.378
17	0.871 (0.825, 0.917)	0.339 (0.218, 0.478)	0.946 (0.913, 0.969)	6.288 (3.406, 11.608)	0.698 (0.578, 0.844)	0.559 (0.379, 0.728)	0.877 (0.834, 0.912)	0.285
18	0.885 (0.845, 0.924)	0.232 (0.130, 0.364)	0.975 (0.949, 0.990)	9.219 (3.851, 22.069)	0.788 (0.681, 0.911)	0.650 (0.408, 0.846)	0.863 (0.820, 0.899)	0.207
19	0.901 (0.860, 0.943)	0.679 (0.540, 0.797)	0.928 (0.891, 0.956)	9.432 (5.960, 14.927)	0.346 (0.236, 0.507)	0.655 (0.519, 0.775)	0.935 (0.899, 0.961)	0.607
20	0.909 (0.871, 0.948)	0.589 (0.450, 0.719)	0.932 (0.895, 0.958)	8.622 (5.303, 14.018)	0.441 (0.322, 0.604)	0.635 (0.490, 0.764)	0.918 (0.880, 0.948)	0.521
21	0.905 (0.865, 0.944)	0.429 (0.297, 0.568)	0.950 (0.917, 0.972)	8.510 (4.702, 15.403)	0.602 (0.479, 0.756)	0.632 (0.460, 0.782)	0.892 (0.851, 0.925)	0.378
22	0.903 (0.867, 0.939)	0.179 (0.089, 0.304)	0.982 (0.959, 0.994)	9.929 (3.529, 27.934)	0.836 (0.740, 0.946)	0.667 (0.384, 0.882)	0.856 (0.812, 0.892)	0.161
23	0.902 (0.861, 0.943)	0.482 (0.347, 0.620)	0.935 (0.900, 0.961)	7.446 (4.415, 12.560)	0.554 (0.429, 0.714)	0.600 (0.443, 0.743)	0.900 (0.859, 0.932)	0.417
24	0.906 (0.867, 0.946)	0.232 (0.130, 0.364)	0.993 (0.974, 0.999)	32.269 (7.488, 39.049)	0.773 (0.669, 0.894)	0.867 (0.595, 0.983)	0.865 (0.823, 0.901)	0.225
25	0.906 (0.866, 0.946)	0.393 (0.265, 0.532)	0.964 (0.935, 0.983)	10.921 (5.477, 21.778)	0.630 (0.510, 0.778)	0.688 (0.500, 0.839)	0.887 (0.846, 0.921)	0.357
26	0.903 (0.863, 0.944)	0.643 (0.504, 0.766)	0.932 (0.895, 0.958)	9.406 (5.844, 15.139)	0.383 (0.269, 0.546)	0.655 (0.514, 0.778)	0.928 (0.891, 0.956)	0.575

***** See Table A1 for specifications of empirical formulas. Areas under the receiver operating characteristic curve (AUC), confident interval (CI), positive likelihood ratio (+LR), negative likelihood ratios (−LR), positive predictive values (+PV), negative predictive values (−PV).

**Table A4:** Prediction performance of the quadratic mixed-effects model without covariance when combined with the 26 estimated fetal weight empirical formulas* in predicting macrosomia.

*Formulas*	AUC (95% CI)	Sensitivity (95% CI)	Specificity (95% CI)	+LR (95% CI)	−LR (95% CI)	+PV (95% CI)	−PV (95% CI)	Youden’s Index
1	0.861 (0.816, 0.906)	0.679 (0.540, 0.797)	0.845 (0.797, 0.886)	4.387 (3.158, 6.094)	0.380 (0.259, 0.558)	0.469 (0.357, 0.583)	0.929 (0.890, 0.957)	0.524
2	0.882 (0.841, 0.923)	0.839 (0.717, 0.924)	0.777 (0.723, 0.825)	3.763 (2.938, 4.820)	0.207 (0.113, 0.378)	0.431 (0.337, 0.530)	0.960 (0.925, 0.982)	0.616
3	0.877 (0.835, 0.919)	0.357 (0.234, 0.496)	0.953 (0.921, 0.975)	7.637 (4.041, 14.454)	0.674 (0.554, 0.821)	0.606 (0.421, 0.771)	0.880 (0.838, 0.915)	0.310
4	0.858 (0.813, 0.903)	0.786 (0.656, 0.884)	0.727 (0.670, 0.778)	2.874 (2.271, 3.637)	0.295 (0.178, 0.489)	0.367 (0.281, 0.459)	0.944 (0.904, 0.971)	0.512
5	0.889 (0.849, 0.930)	0.768 (0.636, 0.870)	0.827 (0.778, 0.870)	4.447 (3.311, 5.980)	0.281 (0.174, 0.453)	0.473 (0.367, 0.580)	0.947 (0.910, 0.971)	0.595
6	0.882 (0.840, 0.923)	0.839 (0.717, 0.924)	0.770 (0.716, 0.818)	3.646 (2.857, 4.651)	0.209 (0.114, 0.381)	0.423 (0.330, 0.521)	0.960 (0.925, 0.981)	0.609
7	0.874 (0.932, 0.917)	0.982 (0.904, 0.999)	0.482 (0.422, 0.542)	1.896 (1.684, 2.135)	0.037 (0.005, 0.259)	0.276 (0.215, 0.344)	0.993 (0.959, 0.999)	0.464
8	0.869 (0.825, 0.913)	0.911 (0.804, 0.970)	0.716 (0.659, 0.768)	3.205 (2.614, 3.929)	0.975 (0.944, 0.992)	0.392 (0.308, 0.482)	0.975 (0.944, 0.992)	0.627
9	0.903 (0.868, 0.938)	0.964 (0.877, 0.996)	0.662 (0.603, 0.717)	2.852 (2.401, 3.387)	0.054 (0.014, 0.211)	0.365 (0.287, 0.448)	0.989 (0.962, 0.999)	0.626
10	0.902 (0.867, 0.937)	0.839 (0.717, 0.924)	0.805 (0.754, 0.851)	4.321 (3.313, 5.634)	0.199 (0.109, 0.364)	0.465 (0.365, 0.567)	0.961 (0.928, 0.982)	0.645
11	0.895 (0.858, 0.932)	0.982 (0.904, 0.999)	0.579 (0.519, 0.638)	2.334 (2.024, 2.691)	0.031 (0.004, 0.216)	0.320 (0.251, 0.395)	0.994 (0.966, 0.999)	0.561
12	0.905 (0.870, 0.939)	0.875 (0.759, 0.948)	0.788 (0.735, 0.834)	4.123 (3.220, 5.279)	0.159 (0.079, 0.318)	0.454 (0.358, 0.552)	0.969 (0.937, 0.987)	0.663
13	0.899 (0.863, 0.935)	0.893 (0.781, 0.960)	0.745 (0.689, 0.795)	3.496 (2.805, 4.357)	0.144 (0.067, 0.307)	0.413 (0.324, 0.506)	0.972 (0.940, 0.990)	0.637
14	0.898 (0.863, 0.934)	0.643 (0.504, 0.766)	0.892 (0.850, 0.926)	5.957 (4.032, 8.801)	0.400 (0.281, 0.570)	0.545 (0.418, 0.669)	0.925 (0.887, 0.954)	0.535
15	0.904 (0.869, 0.939)	0.875 (0.759, 0.948)	0.763 (0.708, 0.811)	3.686 (2.920, 4.652)	0.164 (0.082, 0.329)	0.426 (0.334, 0.522)	0.968 (0.935, 0.987)	0.637
16	0.898 (0.863, 0.934)	0.857 (0.738, 0.936)	0.799 (0.747, 0.844)	4.255 (3.290, 5.504)	0.179 (0.094, 0.341)	0.462 (0.363, 0.562)	0.965 (0.933, 0.985)	0.656
17	0.879 (0.836, 0.921)	0.804 (0.676, 0.898)	0.788 (0.735, 0.834)	3.786 (2.917, 4.915)	0.249 (0.146, 0.425)	0.433 (0.336, 0.533)	0.952 (0.916, 0.976)	0.591
18	0.901 (0.865, 0.938)	0.804 (0.676, 0.898)	0.838 (0.789, 0.879)	4.964 (3.688, 6.682)	0.234 (0.138, 0.399)	0.500 (0.393, 0.607)	0.955 (0.921, 0.977)	0.642
19	0.895 (0.856, 0.933)	0.875 (0.759, 0.948)	0.748 (0.693, 0.798)	3.475 (2.773, 4.354)	0.167 (0.083, 0.335)	0.412 (0.322, 0.506)	0.967 (0.934, 0.987)	0.623
20	0.900 (0.864, 0.936)	0.875 (0.759, 0.948)	0.755 (0.700, 0.805)	3.577 (2.845, 4.498)	0.165 (0.082, 0.332)	0.419 (0.328, 0.514)	0.968 (0.935, 0.987)	0.630
21	0.898 (0.861, 0.935)	0.857 (0.738, 0.936)	0.752 (0.697, 0.801)	3.453 (2.741, 4.350)	0.190 (0.100, 0.362)	0.410 (0.320, 0.505)	0.963 (0.929, 0.984)	0.609
22	0.903 (0.868, 0.938)	0.875 (0.759, 0.948)	0.758 (0.704, 0.808)	3.631 (2.882, 4.574)	0.165 (0.082, 0.330)	0.422 (0.331, 0.518)	0.968 (0.935, 0.987)	0.634
23	0.896 (0.858, 0.934)	0.839 (0.717, 0.924)	0.791 (0.739, 0.838)	4.023 (3.114, 5.197)	0.203 (0.111, 0.371)	0.448 (0.350, 0.548)	0.961 (0.927, 0.982)	0.631
24	0.898 (0.861, 0.935)	0.786 (0.656, 0.884)	0.831 (0.782, 0.873)	4.647 (3.463, 6.238)	0.258 (0.156, 0.427)	0.484 (0.377, 0.591)	0.951 (0.915, 0.974)	0.617
25	0.900 (0.864, 0.936)	0.821 (0.696, 0.911)	0.795 (0.743, 0.841)	4.006 (3.084, 5.205)	0.225 (0.128, 0.395)	0.447 (0.349, 0.548)	0.957 (0.922, 0.979)	0.616
26	0.897 (0.859, 0.934)	0.857 (0.738, 0.936)	0.758 (0.707, 0.808)	3.557 (2.813, 4.496)	0.188 (0.099, 0.359)	0.417 (0.326, 0.513)	0.963 (0.929, 0.984)	0.616

***** See Table A1 for specifications of empirical formulas. Areas under the receiver operating characteristic curve (AUC), confident interval (CI), positive likelihood ratio (+LR), negative likelihood ratios (−LR), positive predictive values (+PV), negative predictive values (−PV).

**Table A5:** Prediction performance of the quadratic mixed-effects model with covariance when combined with the 26 estimated fetal weight empirical formulas* in predicting macrosomia.

*Formulas*	AUC (95% CI)	Sensitivity (95% CI)	Specificity (95% CI)	+LR (95% CI)	−LR (95% CI)	+PV (95% CI)	−PV (95% CI)	Youden’s Index
1	0.860 (0.815, 0.905)	0.661 (0.522, 0.782)	0.845 (0.797, 0.886)	4.272 (3.062, 5.958)	0.401 (0.278, 0.580)	0.463 (0.350, 0.578)	0.925 (0.886, 0.954)	0.506
2	0.882 (0.841, 0.923)	0.839 (0.717, 0.924)	0.781 (0.727, 0.828)	3.825 (2.980, 4.909)	0.206 (0.113, 0.376)	0.435 (0.340, 0.534)	0.960 (0.926, 0.982)	0.620
3	0.877 (0.835, 0.919)	0.357 (0.234, 0.496)	0.953 (0.921, 0.975)	7.637 (4.041, 14.434)	0.674 (0.554, 0.821)	0.606 (0.421, 0.771)	0.880 (0.838, 0.915)	0.310
4	0.857 (0.812, 0.903)	0.786 (0.656, 0.884)	0.730 (0.674, 0.781)	2.912 (2.298, 3.691)	0.293 (0.177, 0.487)	0.370 (0.283, 0.463)	0.944 (0.905, 0.971)	0.516
5	0.889 (0.849, 0.930)	0.768 (0.636, 0.870)	0.827 (0.778, 0.870)	4.447 (3.311, 5.972)	0.281 (0.174, 0.453)	0.473 (0.367, 0.580)	0.947 (0.910, 0.971)	0.595
6	0.881 (0.839, 0.923)	0.839 (0.717, 0.924)	0.770 (0.716, 0.820)	3.646 (2.857, 4.651)	0.209 (0.114, 0.381)	0.423 (0.330, 0.521)	0.960 (0.925, 0.981)	0.609
7	0.873 (0.830, 0.916)	0.982 (0.904, 0.999)	0.478 (0.418, 0.539)	1.883 (1.673, 2.119)	0.037 (0.005, 0.261)	0.275 (0.214, 0.342)	0.993 (0.959, 1.000)	0.461
8	0.868 (0.824, 0.912)	0.911 (0.804, 0.970)	0.712 (0.655, 0.765)	3.165 (2.585, 3.874)	0.125 (0.054, 0.290)	0.389 (0.305, 0.478)	0.975 (0.943, 0.992)	0.623
9	0.902 (0.867, 0.937)	0.964 (0.877, 0.996)	0.669 (0.610, 0.724)	2.914 (2.447, 3.470)	0.053 (0.014, 0.209)	0.370 (0.292, 0.454)	0.989 (0.962, 0.999)	0.633
10	0.902 (0.867, 0.937)	0.839 (0.717, 0.924)	0.806 (0.754, 0.851)	4.321 (3.313, 5.634)	0.199 (0.109, 0.364)	0.465 (0.365, 0.567)	0.961 (0.928, 0.982)	0.645
11	0.893 (0.856, 0.931)	0.982 (0.904, 0.999)	0.579 (0.519, 0.638)	2.334 (2.024, 2.691)	0.031 (0.004, 0.216)	0.320 (0.251, 0.395)	0.994 (0.966, 1.000)	0.561
12	0.905 (0.870, 0.939)	0.875 (0.759, 0.948)	0.784 (0.731, 0.831)	4.054 (3.173, 5.179)	0.159 (0.079, 0.320)	0.500 (0.354, 0.548)	0.969 (0.937, 0.987)	0.659
13	0.898 (0.862, 0.934)	0.893 (0.781, 0.960)	0.748 (0.693, 0.798)	3.546 (2.840, 4.427)	0.143 (0.067, 0.306)	0.417 (0.327, 0.510)	0.972 (0.940, 0.990)	0.641
14	0.898 (0.875, 0.948)	0.643 (0.504, 0.766)	0.892 (0.850, 0.926)	5.957 (4.032, 8.801)	0.400 (0.281, 0.570)	0.545 (0.418, 0.669)	0.925 (0.887, 0.954)	0.535
15	0.903 (0.869, 0.938)	0.875 (0.759, 0.948)	0.759 (0.704, 0.808)	3.631 (2.882, 4.574)	0.165 (0.082, 0.330)	0.422 (0.331, 0.518)	0.968 (0.935, 0.987)	0.634
16	0.898 (0.862, 0.934)	0.857 (0.738, 0.936)	0.799 (0.747, 0.844)	4.255 (3.290, 5.504)	0.179 (0.094, 0.341)	0.462 (0.363, 0.562)	0.965 (0.933, 0.985)	0.656
17	0.878 (0.836, 0.921)	0.786 (0.656, 0.884)	0.788 (0.735, 0.834)	3.702 (2.842, 4.823)	0.272 (0.164, 0.451)	0.427 (0.330, 0.528)	0.948 (0.911, 0.973)	0.573
18	0.901 (0.864, 0.938)	0.804 (0.676, 0.898)	0.842 (0.793, 0.883)	5.077 (3.760, 6.856)	0.233 (0.137, 0.397)	0.506 (0.398, 0.613)	0.955 (0.921, 0.977)	0.645
19	0.894 (0.856, 0.932)	0.875 (0.759, 0.948)	0.745 (0.689, 0.795)	3.426 (2.739, 4.285)	0.168 (0.084, 0.337)	0.408 (0.320, 0.502)	0.967 (0.934, 0.987)	0.620
20	0.900 (0.865, 0.936)	0.875 (0.759, 0.948)	0.755 (0.700, 0.805)	3.577 (2.845, 4.498)	0.165 (0.082, 0.332)	0.419 (0.328, 0.514)	0.968 (0.935, 0.987)	0.630
21	0.897 (0.860, 0.934)	0.857 (0.738, 0.936)	0.755 (0.700, 0.805)	3.504 (2.777, 4.422)	0.189 (0.099, 0.360)	0.414 (0.323, 0.509)	0.963 (0.929, 0.984)	0.613
22	0.903 (0.868, 0.938)	0.875 (0.759, 0.948)	0.755 (0.700, 0.805)	3.577 (2.845, 4.498)	0.165 (0.082, 0.332)	0.419 (0.328, 0.514)	0.968 (0.935, 0.987)	0.630
23	0.895 (0.857, 0.933)	0.821 (0.696, 0.911)	0.791 (0.739, 0.838)	3.937 (3.037, 5.104)	0.226 (0.128, 0.397)	0.442 (0.345, 0.543)	0.957 (0.921, 0.979)	0.613
24	0.898 (0.861, 0.935)	0.786 (0.656, 0.884)	0.831 (0.782, 0.873)	4.647 (3.463, 6.238)	0.258 (0.156, 0.427)	0.484 (0.377, 0.591)	0.951 (0.915, 0.974)	0.617
25	0.899 (0.863, 0.936)	0.821 (0.696, 0.911)	0.799 (0.747, 0.844)	4.078 (3.132, 5.310)	0.957 (0.922, 0.979)	0.451 (0.352, 0.553)	0.957 (0.922, 0.979)	0.620
26	0.897 (0.859, 0.934)	0.857 (0.738, 0.936)	0.755 (0.700, 0.805)	3.504 (2.777, 4.422)	0.189 (0.099, 0.360)	0.414 (0.323, 0.509)	0.963 (0.929, 0.984)	0.613

***** See Table A1 for specifications of empirical formulas. Areas under the receiver operating characteristic curve (AUC), confident interval (CI), positive likelihood ratio (+LR), negative likelihood ratios (−LR), positive predictive values (+PV), negative predictive values (−PV).

**Table A6:** Prediction performance of the three-parameter mixed-effects logistic model without covariance when combined with the 26 estimated fetal weight empirical formulas* in predicting large for gestational age.

*Formulas*	AUC (95% CI)	Sensitivity (95% CI)	Specificity (95% CI)	+LR (95% CI)	−LR (95% CI)	+PV (95% CI)	−PV (95% CI)	Youden’s Index
1	0.865 (0.810, 0.919)	0.424 (0.255, 0.608)	0.930 (0.895, 0.956)	6.081 (3.429, 10.783)	0.612 (0.461, 0.831)	0.400 (0.239, 0.579)	0.936 (0.903, 0.961)	0.354
2	0.867 (0.812, 0.922)	0.455 (0.281, 0.636)	0.940 (0.907, 0.964)	7.601 (4.241, 13.622)	0.580 (0.424, 0.793)	0.454 (0.281, 0.636)	0.940 (0.907, 0.964)	0.395
3	0.867 (0.811, 0.923)	0.485 (0.308, 0.665)	0.940 (0.907, 0.964)	8.108 (4.587, 14.330)	0.548 (0.393, 0.764)	0.471 (0.298, 0.649)	0.943 (0.911, 0.967)	0.425
4	0.868 (0.814, 0.922)	0.455 (0.281, 0.636)	0.940 (0.907, 0.964)	7.601 (4.241, 13.622)	0.580 (0.424, 0.793)	0.454 (0.281, 0.636)	0.940 (0.907, 0.964)	0.395
5	0.868 (0.812, 0.923)	0.455 (0.281, 0.636)	0.944 (0.911, 0.967)	8.048 (4.443, 14.578)	0.578 (0.423, 0.790)	0.469 (0.291, 0.653)	0.940 (0.907, 0.964)	0.398
6	0.873 (0.818, 0.927)	0.515 (0.335, 0.692)	0.937 (0.903, 0.962)	8.161 (4.724, 14.100)	0.518 (0.364, 0.737)	0.472 (0.304, 0.645)	0.946 (0.914, 0.969)	0.452
7	0.891 (0.839, 0.944)	0.455 (0.281, 0.636)	0.937 (0.903, 0.962)	7.201 (4.057, 12.780)	0.582 (0.426, 0.796)	0.441 (0.272, 0.621)	0.940 (0.907, 0.964)	0.391
8	0.876 (0.820, 0.932)	0.485 (0.308, 0.665)	0.944 (0.911, 0.967)	8.585 (4.805, 15.339)	0.546 (0.392, 0.761)	0.485 (0.308, 0.665)	0.944 (0.911, 0.967)	0.428
9	0.894 (0.854, 0.934)	0.455 (0.281, 0.636)	0.934 (0.899, 0.959)	6.841 (3.889, 12.034)	0.584 (0.427, 0.780)	0.429 (0.263, 0.606)	0.940 (0.907, 0.964)	0.388
10	0.886 (0.846, 0.927)	0.485 (0.308, 0.665)	0.930 (0.895, 0.956)	6.949 (4.041, 11.950)	0.554 (0.397, 0.772)	0.432 (0.271, 0.605)	0.943 (0.910, 0.966)	0.415
11	0.895 (0.856, 0.935)	0.485 (0.308, 0.665)	0.940 (0.907, 0.964)	8.108 (4.587, 14.330)	0.548 (0.393, 0.764)	0.471 (0.298, 0.649)	0.943 (0.911, 0.967)	0.425
12	0.891 (0.849, 0.933)	0.515 (0.335, 0.692)	0.950 (0.919, 0.972)	10.337 (5.707, 18.724)	0.510 (0.359, 0.726)	0.531 (0.347, 0.709)	0.947 (0.915, 0.969)	0.465
13	0.890 (0.849, 0.932)	0.485 (0.308, 0.665)	0.944 (0.911, 0.967)	8.585 (4.805, 15.339)	0.546 (0.392, 0.761)	0.485 (0.308, 0.665)	0.944 (0.911, 0.967)	0.428
14	0.886 (0.845, 0.926)	0.545 (0.364, 0.719)	0.944 (0.911, 0.967)	9.658 (5.533, 16.856)	0.482 (0.331, 0.701)	0.514 (0.340, 0.686)	0.950 (0.919, 0.972)	0.489
15	0.892 (0.852, 0.931)	0.485 (0.308, 0.665)	0.940 (0.907, 0.964)	8.108 (4.587, 14.330)	0.548 (0.393, 0.764)	0.471 (0.298, 0.649)	0.943 (0.911, 0.967)	0.425
16	0.883 (0.842, 0.924)	0.485 (0.308, 0.665)	0.937 (0.903, 0.962)	7.681 (4.389, 13.441)	0.550 (0.394, 0.767)	0.457 (0.288, 0.634)	0.943 (0.911, 0.967)	0.422
17	0.861 (0.803, 0.919)	0.455 (0.281, 0.636)	0.934 (0.899, 0.959)	6.841 (3.889, 12.034)	0.584 (0.427, 0.780)	0.429 (0.263, 0.606)	0.940 (0.907, 0.964)	0.388
18	0.871 (0.818, 0.924)	0.515 (0.335, 0.692)	0.937 (0.903, 0.962)	8.161 (4.724, 14.100)	0.518 (0.364, 0.737)	0.472 (0.304, 0.645)	0.946 (0.914, 0.969)	0.452
19	0.887 (0.841, 0.933)	0.515 (0.335, 0.692)	0.937 (0.903, 0.962)	8.161 (4.724, 14.100)	0.518 (0.364, 0.737)	0.472 (0.304, 0.645)	0.946 (0.914, 0.969)	0.452
20	0.892 (0.853, 0.932)	0.545 (0.364, 0.719)	0.944 (0.911, 0.967)	9.658 (5.533, 16.856)	0.482 (0.331, 0.701)	0.514 (0.340, 0.686)	0.950 (0.919, 0.972)	0.489
21	0.890 (0.850, 0.931)	0.424 (0.255, 0.608)	0.937 (0.903, 0.962)	6.721 (3.728, 12.117)	0.615 (0.458, 0.825)	0.424 (0.255, 0.608)	0.937 (0.903, 0.962)	0.361
22	0.884 (0.840, 0.929)	0.485 (0.308, 0.665)	0.940 (0.907, 0.964)	8.108 (4.587, 14.330)	0.548 (0.393, 0.764)	0.471 (0.298, 0.649)	0.943 (0.911, 0.967)	0.425
23	0.882 (0.836, 0.929)	0.485 (0.308, 0.665)	0.940 (0.907, 0.964)	8.108 (4.587, 14.330)	0.548 (0.393, 0.764)	0.471 (0.298, 0.649)	0.943 (0.911, 0.967)	0.425
24	0.887 (0.846, 0.929)	0.455 (0.281, 0.636)	0.937 (0.903, 0.962)	7.201 (4.057, 12.780)	0.582 (0.426, 0.796)	0.441 (0.272, 0.621)	0.940 (0.907, 0.964)	0.391
25	0.889 (0.847, 0.931)	0.455 (0.281, 0.636)	0.937 (0.903, 0.962)	7.201 (4.057, 12.780)	0.582 (0.426, 0.796)	0.441 (0.272, 0.621)	0.940 (0.907, 0.964)	0.391
26	0.885 (0.840, 0.930)	0.515 (0.335, 0.692)	0.940 (0.907, 0.964)	8.614 (4.936, 15.035)	0.516 (0.362, 0.734)	0.456 (0.243, 0.656)	0.946 (0.915, 0.969)	0.455

***** See Table A1 for specifications of empirical formulas. Areas under the receiver operating characteristic curve (AUC), confident interval (CI), positive likelihood ratio (+LR), negative likelihood ratios (−LR), positive predictive values (+PV), negative predictive values (−PV).

**Table A7:** Prediction performance of the three-parameter mixed-effects logistic model with covariance when combined with the 26 estimated fetal weight empirical formulas* in predicting large for gestational age.

*Formulas*	AUC (95% CI)	Sensitivity (95% CI)	Specificity (95% CI)	+LR (95% CI)	−LR (95% CI)	+PV (95% CI)	−PV (95% CI)	Youden’s index
1	0.868 (0.814, 0.922)	0.455 (0.281, 0.636)	0.934 (0.899, 0.959)	6.841 (3.889, 12.034)	0.584 (0.427, 0.799)	0.429 (0.263, 0.606)	0.940 (0.907, 0.964)	0.388
2	0.867 (0.812, 0.922)	0.455 (0.281, 0.636)	0.940 (0.907, 0.964)	7.601 (4.241, 13.622)	0.580 (0.424, 0.793)	0.454 (0.281, 0.636)	0.940 (0.907, 0.964)	0.395
3	0.867 (0.811, 0.923)	0.515 (0.335, 0.692)	0.940 (0.907, 0.964)	8.614 (4.936, 15.035)	0.516 (0.362, 0.734)	0.456 (0.243, 0.656)	0.946 (0.915, 0.969)	0.455
4	0.868 (0.814, 0.922)	0.455 (0.281, 0.636)	0.937 (0.903, 0.962)	7.201 (4.057, 12.780)	0.582 (0.426, 0.796)	0.441 (0.272, 0.621)	0.940 (0.907, 0.964)	0.391
5	0.868 (0.812, 0.923)	0.455 (0.281, 0.636)	0.940 (0.907, 0.964)	7.601 (4.241, 13.622)	0.580 (0.424, 0.793)	0.454 (0.281, 0.636)	0.940 (0.907, 0.964)	0.395
6	0.873 (0.818, 0.927)	0.545 (0.364, 0.719)	0.944 (0.911, 0.967)	9.658 (5.533, 16.856)	0.482 (0.331, 0.701)	0.514 (0.340, 0.686)	0.950 (0.919, 0.972)	0.489
7	0.891 (0.839, 0.944)	0.455 (0.281, 0.636)	0.940 (0.907, 0.964)	7.601 (4.241, 13.622)	0.580 (0.424, 0.793)	0.454 (0.281, 0.636)	0.940 (0.907, 0.964)	0.395
8	0.876 (0.820, 0.932)	0.485 (0.308, 0.665)	0.947 (0.915, 0.969)	9.121 (5.044, 16.495)	0.544 (0.390, 0.758)	0.500 (0.319, 0.681)	0.944 (0.911, 0.967)	0.432
9	0.894 (0.854, 0.934)	0.455 (0.281, 0.636)	0.937 (0.903, 0.962)	7.201 (4.057, 12.780)	0.582 (0.426, 0.796)	0.441 (0.272, 0.621)	0.940 (0.907, 0.964)	0.391
10	0.886 (0.846, 0.927)	0.455 (0.281, 0.636)	0.934 (0.899, 0.959)	6.841 (3.889, 12.034)	0.584 (0.427, 0.799)	0.429 (0.263, 0.606)	0.940 (0.907, 0.964)	0.388
11	0.895 (0.856, 0.935)	0.485 (0.308, 0.665)	0.940 (0.907, 0.964)	8.108 (4.587, 14.330)	0.548 (0.393, 0.764)	0.471 (0.298, 0.649)	0.943 (0.911, 0.967)	0.425
12	0.891 (0.849, 0.933)	0.515 (0.335, 0.692)	0.950 (0.919, 0.972)	10.337 (5.707, 18.724)	0.510 (0.359, 0.726)	0.465 (0.255, 0.664)	0.947 (0.915, 0.969)	0.465
13	0.890 (0.849, 0.932)	0.455 (0.281, 0.636)	0.940 (0.907, 0.964)	7.601 (4.241, 13.622)	0.580 (0.424, 0.793)	0.454 (0.281, 0.636)	0.940 (0.907, 0.964)	0.395
14	0.886 (0.845, 0.926)	0.485 (0.308, 0.665)	0.940 (0.907, 0.964)	8.108 (4.587, 14.330)	0.548 (0.393, 0.764)	0.471 (0.298, 0.649)	0.943 (0.911, 0.967)	0.425
15	0.892 (0.852, 0.931)	0.455 (0.281, 0.636)	0.944 (0.911, 0.967)	8.048 (4.443, 14.578)	0.578 (0.423, 0.790)	0.469 (0.291, 0.653)	0.940 (0.907, 0.964)	0.398
16	0.883 (0.842, 0.924)	0.485 (0.308, 0.665)	0.937 (0.903, 0.962)	7.681 (4.389, 13.441)	0.550 (0.394, 0.767)	0.457 (0.288, 0.634)	0.943 (0.911, 0.967)	0.422
17	0.861 (0.803, 0.919)	0.424 (0.255, 0.608)	0.937 (0.903, 0.962)	6.721 (4.728, 12.117)	0.615 (0.458, 0.825)	0.424 (0.255, 0.608)	0.937 (0.903, 0.962)	0.361
18	0.871 (0.818, 0.924)	0.455 (0.281, 0.636)	0.934 (0.899, 0.959)	6.841 (3.889, 12.034)	0.584 (0.427, 0.780)	0.429 (0.263, 0.606)	0.940 (0.907, 0.964)	0.388
19	0.887 (0.841, 0.933)	0.485 (0.308, 0.665)	0.940 (0.907, 0.964)	8.108 (4.587, 14.330)	0.548 (0.393, 0.764)	0.471 (0.298, 0.649)	0.943 (0.911, 0.967)	0.425
20	0.892 (0.853, 0.932)	0.515 (0.335, 0.692)	0.944 (0.911, 0.967)	9.121 (5.168, 16.099)	0.514 (0.361, 0.731)	0.500 (0.247, 0.659)	0.947 (0.915, 0.969)	0.459
21	0.890 (0.850, 0.931)	0.455 (0.281, 0.636)	0.937 (0.903, 0.962)	7.201 (4.057, 12.780)	0.582 (0.426, 0.796)	0.441 (0.272, 0.621)	0.940 (0.907, 0.964)	0.391
22	0.884 (0.840, 0.929)	0.545 (0.364, 0.719)	0.944 (0.911, 0.967)	9.658 (5.533, 16.846)	0.482 (0.331, 0.701)	0.514 (0.340, 0.686)	0.950 (0.919, 0.972)	0.489
23	0.882 (0.836, 0.929)	0.485 (0.308, 0.665)	0.940 (0.907, 0.964)	8.108 (4.587, 14.330)	0.548 (0.393, 0.764)	0.471 (0.298, 0.649)	0.943 (0.911, 0.967)	0.425
24	0.887 (0.846, 0.929)	0.424 (0.255, 0.608)	0.934 (0.899, 0.959)	6.385 (3.572, 11.412)	0.617 (0.459, 0.828)	0.412 (0.246, 0.593)	0.937 (0.903, 0.961)	0.358
25	0.889 (0.847, 0.931)	0.485 (0.308, 0.665)	0.937 (0.903, 0.962)	7.681 (4.389, 13.441)	0.550 (0.394, 0.767)	0.457 (0.288, 0.634)	0.943 (0.911, 0.967)	0.422
26	0.885 (0.840, 0.930)	0.485 (0.308, 0.665)	0.940 (0.907, 0.964)	8.108 (4.587, 14.330)	0.548 (0.393, 0.764)	0.471 (0.298, 0.649)	0.943 (0.911, 0.967)	0.425

***** See Table A1 for specifications of empirical formulas. Areas under the receiver operating characteristic curve (AUC), confident interval (CI), positive likelihood ratio (+LR), negative likelihood ratios (−LR), positive predictive values (+PV), negative predictive values (−PV).

**Table A8:** Prediction performance of the quadratic mixed-effects model without covariance when combined with the 26 estimated fetal weight empirical formulas* in predicting large for gestational age.

*Formulas*	AUC (95% CI)	Sensitivity (95% CI)	Specificity (95% CI)	+LR (95% CI)	−LR (95% CI)	+PV (95% CI)	−PV (95% CI)	Youden’s Index
1	0.864 (0.810, 0.919)	0.455 (0.281, 0.636)	0.937 (0.903, 0.962)	7.201 (4.057, 12.780)	0.582 (0.426, 0.796)	0.441 (0.272, 0.621)	0.940 (0.907, 0.964)	0.391
2	0.867 (0.812, 0.922)	0.455 (0.281, 0.636)	0.937 (0.903, 0.962)	7.201 (4.057, 12.780)	0.582 (0.426, 0.796)	0.441 (0.272, 0.621)	0.940 (0.907, 0.964)	0.391
3	0.867 (0.811, 0.923)	0.455 (0.281, 0.636)	0.937 (0.903, 0.962)	7.201 (4.057, 12.780)	0.582 (0.426, 0.796)	0.441 (0.272, 0.621)	0.940 (0.907, 0.964)	0.391
4	0.868 (0.814, 0.922)	0.485 (0.308, 0.665)	0.940 (0.907, 0.964)	8.108 (4.587, 14.330)	0.548 (0.393, 0.764)	0.471 (0.298, 0.649)	0.943 (0.911, 0.967)	0.425
5	0.868 (0.812, 0.923)	0.455 (0.281, 0.636)	0.937 (0.903, 0.962)	7.201 (4.057, 12.780)	0.582 (0.426, 0.796)	0.441 (0.272, 0.621)	0.940 (0.907, 0.964)	0.391
6	0.873 (0.818, 0.927)	0.576 (0.392, 0.745)	0.950 (0.919, 0.972)	11.554 (6.510, 20.505)	0.446 (0.300, 0.665)	0.559 (0.379, 0.728)	0.953 (0.923, 0.974)	0.526
7	0.891 (0.839, 0.944)	0.455 (0.281, 0.636)	0.937 (0.903, 0.962)	7.201 (4.057, 12.780)	0.582 (0.426, 0.796)	0.441 (0.272, 0.621)	0.940 (0.907, 0.964)	0.391
8	0.876 (0.820, 0.932)	0.545 (0.364, 0.719)	0.947 (0.915, 0.969)	10.261 (5.806, 18.136)	0.480 (0.330, 0.698)	0.529 (0.351, 0.702)	0.950 (0.919, 0.972)	0.492
9	0.894 (0.854, 0.934)	0.455 (0.281, 0.636)	0.937 (0.903, 0.962)	7.201 (4.057, 12.780)	0.582 (0.426, 0.796)	0.441 (0.272, 0.621)	0.940 (0.907, 0.964)	0.391
10	0.886 (0.846, 0.927)	0.455 (0.281, 0.636)	0.937 (0.903, 0.962)	7.201 (4.057, 12.780)	0.582 (0.426, 0.796)	0.441 (0.272, 0.621)	0.940 (0.907, 0.964)	0.391
11	0.895 (0.856, 0.935)	0.455 (0.281, 0.636)	0.937 (0.903, 0.962)	7.201 (4.057, 12.780)	0.582 (0.426, 0.796)	0.441 (0.272, 0.621)	0.940 (0.907, 0.964)	0.391
12	0.891 (0.849, 0.933)	0.515 (0.335, 0.692)	0.944 (0.911, 0.967)	9.121 (5.168, 16.099)	0.514 (0.361, 0.731)	0.500 (0.247, 0.659)	0.947 (0.915, 0.969)	0.459
13	0.890 (0.849, 0.932)	0.485 (0.308, 0.665)	0.940 (0.907, 0.964)	8.108 (4.587, 14.330)	0.548 (0.393, 0.764)	0.471 (0.298, 0.649)	0.943 (0.911, 0.967)	0.425
14	0.886 (0.845, 0.926)	0.424 (0.255, 0.608)	0.930 (0.895, 0.956)	6.081 (3.429, 10.783)	0.619 (0.461, 0.831)	0.400 (0.239, 0.579)	0.936 (0.903, 0.961)	0.354
15	0.892 (0.852, 0.931)	0.485 (0.308, 0.665)	0.940 (0.907, 0.964)	8.108 (4.587, 14.330)	0.548 (0.393, 0.764)	0.471 (0.298, 0.649)	0.943 (0.911, 0.967)	0.425
16	0.883 (0.842, 0.924)	0.455 (0.281, 0.636)	0.937 (0.903, 0.962)	7.201 (4.057, 12.780)	0.582 (0.426, 0.796)	0.441 (0.272, 0.621)	0.940 (0.907, 0.964)	0.391
17	0.861 (0.803, 0.919)	0.485 (0.308, 0.665)	0.940 (0.907, 0.964)	8.108 (4.587, 14.330)	0.548 (0.393, 0.764)	0.471 (0.298, 0.649)	0.943 (0.911, 0.967)	0.425
18	0.871 (0.818, 0.924)	0.485 (0.308, 0.665)	0.943 (0.911, 0.967)	8.585 (4.805, 15.339)	0.546 (0.392, 0.761)	0.485 (0.308, 0.665)	0.943 (0.911, 0.967)	0.428
19	0.887 (0.841, 0.933)	0.515 (0.335, 0.692)	0.944 (0.911, 0.967)	9.121 (5.168, 16.099)	0.514 (0.361, 0.731)	0.500 (0.247, 0.659)	0.947 (0.915, 0.969)	0.459
20	0.892 (0.853, 0.932)	0.455 (0.281, 0.636)	0.937 (0.903, 0.962)	7.201 (4.057, 12.780)	0.582 (0.426, 0.796)	0.441 (0.272, 0.621)	0.940 (0.907, 0.964)	0.391
21	0.890 (0.850, 0.931)	0.485 (0.308, 0.665)	0.937 (0.903, 0.962)	7.681 (4.389, 13.441)	0.550 (0.394, 0.767)	0.457 (0.288, 0.634)	0.943 (0.911, 0.967)	0.422
22	0.884 (0.840, 0.929)	0.485 (0.308, 0.665)	0.940 (0.907, 0.964)	8.108 (4.587, 14.330)	0.548 (0.393, 0.764)	0.471 (0.298, 0.649)	0.943 (0.911, 0.967)	0.425
23	0.882 (0.836, 0.929)	0.485 (0.308, 0.665)	0.940 (0.907, 0.964)	8.108 (4.587, 14.330)	0.548 (0.393, 0.764)	0.471 (0.298, 0.649)	0.943 (0.911, 0.967)	0.425
24	0.887 (0.846, 0.929)	0.485 (0.308, 0.665)	0.940 (0.907, 0.964)	8.108 (4.587, 14.330)	0.548 (0.393, 0.764)	0.471 (0.298, 0.649)	0.943 (0.911, 0.967)	0.425
25	0.889 (0.847, 0.931)	0.515 (0.335, 0.692)	0.940 (0.907, 0.964)	8.614 (4.936, 15.035)	0.516 (0.362, 0.734)	0.456 (0.243, 0.656)	0.946 (0.915, 0.969)	0.455
26	0.885 (0.840, 0.930)	0.485 (0.308, 0.665)	0.940 (0.907, 0.964)	8.108 (4.587, 14.330)	0.548 (0.393, 0.764)	0.471 (0.298, 0.649)	0.943 (0.911, 0.967)	0.425

***** See Table A1 for specifications of empirical formulas. Areas under the receiver operating characteristic curve (AUC), confident interval (CI), positive likelihood ratio (+LR), negative likelihood ratios (−LR), positive predictive values (+PV), negative predictive values (−PV).

**Table A9:** Prediction performance of the quadratic mixed-effects model with covariance when combined with the 26 estimated fetal weight empirical formulas* in predicting large for gestational age.

*Formulas*	AUC (95% CI)	Sensitivity (95% CI)	Specificity (95% CI)	+LR (95% CI)	−LR (95% CI)	+PV (95% CI)	−PV (95% CI)	Youden’s index
1	0.865 (0.810, 0.919)	0.455 (0.281, 0.636)	0.937 (0.903, 0.962)	7.201 (4.057, 12.780)	0.582 (0.426, 0.796)	0.441 (0.272, 0.621)	0.940 (0.907, 0.964)	0.391
2	0.867 (0.812, 0.922)	0.455 (0.281, 0.636)	0.937 (0.903, 0.962)	7.201 (4.057, 12.780)	0.582 (0.426, 0.796)	0.441 (0.272, 0.621)	0.940 (0.907, 0.964)	0.391
3	0.867 (0.811, 0.923)	0.455 (0.281, 0.636)	0.934 (0.899, 0.959)	6.841 (3.889, 12.034)	0.584 (0.427, 0.799)	0.429 (0.263, 0.606)	0.940 (0.907, 0.964)	0.388
4	0.868 (0.814, 0.922)	0.485 (0.308, 0.665)	0.937 (0.903, 0.962)	7.681 (4.389, 13.441)	0.550 (0.394, 0.767)	0.457 (0.288, 0.634)	0.943 (0.911, 0.967)	0.422
5	0.868 (0.812, 0.923)	0.455 (0.281, 0.636)	0.937 (0.903, 0.962)	7.201 (4.057, 12.780)	0.582 (0.426, 0.796)	0.441 (0.272, 0.621)	0.940 (0.907, 0.964)	0.391
6	0.873 (0.818, 0.927)	0.576 (0.392, 0.745)	0.950 (0.919, 0.972)	11.554 (6.510, 20.505)	0.446 (0.300, 0.665)	0.559 (0.379, 0.728)	0.953 (0.923, 0.974)	0.526
7	0.891 (0.839, 0.944)	0.455 (0.281, 0.636)	0.937 (0.903, 0.962)	7.201 (4.057, 12.780)	0.582 (0.426, 0.796)	0.441 (0.272, 0.621)	0.940 (0.907, 0.964)	0.391
8	0.876 (0.820, 0.932)	0.515 (0.335, 0.692)	0.947 (0.915, 0.969)	9.691 (5.424, 17.316)	0.512 (0.360, 0.727)	0.515 (0.335, 0.692)	0.947 (0.915, 0.969)	0.462
9	0.894 (0.854, 0.934)	0.455 (0.281, 0.636)	0.937 (0.903, 0.962)	7.201 (4.057, 12.780)	0.582 (0.426, 0.796)	0.441 (0.272, 0.621)	0.940 (0.907, 0.964)	0.391
10	0.886 (0.846, 0.927)	0.455 (0.281, 0.636)	0.937 (0.903, 0.962)	7.201 (4.057, 12.780)	0.582 (0.426, 0.796)	0.441 (0.272, 0.621)	0.940 (0.907, 0.964)	0.391
11	0.895 (0.856, 0.935)	0.455 (0.281, 0.636)	0.937 (0.903, 0.962)	7.201 (4.057, 12.780)	0.582 (0.426, 0.796)	0.441 (0.272, 0.621)	0.940 (0.907, 0.964)	0.391
12	0.891 (0.849, 0.933)	0.515 (0.335, 0.692)	0.944 (0.911, 0.967)	9.121 (5.168, 16.099)	0.514 (0.361, 0.731)	0.500 (0.247, 0.659)	0.947 (0.915, 0.969)	0.459
13	0.890 (0.849, 0.932)	0.485 (0.308, 0.665)	0.940 (0.907, 0.964)	8.108 (4.587, 14.330)	0.548 (0.393, 0.764)	0.471 (0.298, 0.649)	0.943 (0.911, 0.967)	0.425
14	0.886 (0.845, 0.926)	0.424 (0.255, 0.608)	0.930 (0.895, 0.956)	6.081 (3.429, 10.783)	0.619 (0.461, 0.831)	0.400 (0.239, 0.579)	0.936 (0.903, 0.961)	0.354
15	0.892 (0.852, 0.931)	0.455 (0.281, 0.636)	0.940 (0.907, 0.964)	7.601 (4.241, 13.622)	0.580 (0.424, 0.793)	0.454 (0.281, 0.636)	0.940 (0.907, 0.964)	0.395
16	0.883 (0.842, 0.924)	0.455 (0.281, 0.636)	0.937 (0.903, 0.962)	7.201 (4.057, 12.780)	0.582 (0.426, 0.796)	0.441 (0.272, 0.621)	0.940 (0.907, 0.964)	0.391
17	0.861 (0.803, 0.919)	0.455 (0.281, 0.636)	0.937 (0.903, 0.962)	7.201 (4.057, 12.780)	0.582 (0.426, 0.796)	0.441 (0.272, 0.621)	0.940 (0.907, 0.964)	0.391
18	0.871 (0.818, 0.924)	0.485 (0.308, 0.665)	0.944 (0.911, 0.967)	8.585 (4.805, 15.339)	0.546 (0.392, 0.761)	0.485 (0.308, 0.665)	0.944 (0.911, 0.967)	0.428
19	0.887 (0.841, 0.933)	0.515 (0.335, 0.692)	0.944 (0.911, 0.967)	9.121 (5.168, 16.099)	0.514 (0.361, 0.731)	0.500 (0.247, 0.659)	0.947 (0.915, 0.969)	0.459
20	0.892 (0.853, 0.932)	0.455 (0.281, 0.636)	0.940 (0.907, 0.964)	7.601 (4.241, 13.622)	0.580 (0.424, 0.793)	0.454 (0.281, 0.636)	0.940 (0.907, 0.964)	0.395
21	0.890 (0.850, 0.931)	0.485 (0.308, 0.665)	0.937 (0.903, 0.962)	7.681 (4.389, 13.441)	0.550 (0.394, 0.767)	0.457 (0.288, 0.634)	0.943 (0.911, 0.967)	0.422
22	0.884 (0.840, 0.929)	0.485 (0.308, 0.665)	0.940 (0.907, 0.964)	8.108 (4.587, 14.330)	0.548 (0.393, 0.764)	0.471 (0.298, 0.649)	0.943 (0.911, 0.967)	0.425
23	0.882 (0.836, 0.929)	0.485 (0.308, 0.665)	0.940 (0.907, 0.964)	8.108 (4.587, 14.330)	0.548 (0.393, 0.764)	0.471 (0.298, 0.649)	0.943 (0.911, 0.967)	0.425
24	0.887 (0.846, 0.929)	0.485 (0.308, 0.665)	0.940 (0.907, 0.964)	8.108 (4.587, 14.330)	0.548 (0.393, 0.764)	0.471 (0.298, 0.649)	0.943 (0.911, 0.967)	0.425
25	0.889 (0.847, 0.931)	0.515 (0.335, 0.692)	0.944 (0.911, 0.967)	9.121 (5.168, 16.099)	0.514 (0.361, 0.731)	0.500 (0.247, 0.659)	0.947 (0.915, 0.969)	0.459
26	0.885 (0.840, 0.930)	0.485 (0.308, 0.665)	0.940 (0.907, 0.964)	8.108 (4.587, 14.330)	0.548 (0.393, 0.764)	0.471 (0.298, 0.649)	0.943 (0.911, 0.967)	0.425

***** See Table A1 for specifications of empirical formulas. Areas under the receiver operating characteristic curve (AUC), confident interval (CI), positive likelihood ratio (+LR), negative likelihood ratios (−LR), positive.
